# The complete chloroplast genome of *Epimedium sutchuenense* Franch. (Berberidaceae)

**DOI:** 10.1080/23802359.2021.1973923

**Published:** 2021-10-23

**Authors:** Xiang Liu, Yixin Zhang, Cheng Zhang, Chaoqun Xu, Weihan Qin, Guoan Shen, Baolin Guo

**Affiliations:** a Institute of Medicinal Plant Development, Chinese Academy of Medical Science, Peking Union Medical College, Beijing, China; b Chongqing Academy of Chinese Materia Medica, Chongqing, China

**Keywords:** Chloroplast genome, *Epimedium sutchuenense*, Berberidaceae, infrageneric classification, phylogenetic relationship

## Abstract

*Epimedium* L. is the largest herbaceous genus in the family Berberidaceae which comprises more than 60 species. *Epimedium sutchuenense* Franch. is narrowly inhabited in the Daba Mountains of China. In the current study, we assembled the first complete chloroplast genome of *E. sutchuenense* through Illumina paired-end sequencing. The complete chloroplast genome of *E. sutchuenense* was 157,218 bp in length and the total GC content was 38.78%. A total of 112 unique genes were identified, including 78 protein-coding genes, 30 tRNA genes and 4 rRNA genes. The phylogenetic analysis demonstrated that *E. sutchuenense* was sister to *Epimedium wushanense* T. S. Ying. Our results provided valuable information for further phylogenetic research and germplasm exploration of *Epimedium* genus.

*Epimedium* L. is the largest herbaceous genus belonging to the family Berberidaceae with more than 60 perennial plant species discontinuously distributed from North Africa (Algeria) to East Asia (Stearn 2002; Ying 2002). Since more than 50 *Epimedium* species had been discovered in China, covering more than 80 percent of this genus, China is now considered to be the modern diversity center of *Epimedium* species (De Smet et al. 2012). The leaves of *Epimedium* plants had long been used as traditional Chinese medicine ‘Herba Epimedii’ for their special effects of nourishing kidney, muscles, and bones. Prenylated flavonol glycosides (such as Icariin, epimedii A, B, and C), as the main components of Herba Epimedii, has been verified to possess wide-reaching bioactive activities such as regulating bone modeling, anti-tumor, anti-aging, etc. (Liu et al. 2006; Wu et al. 2003; Ma et al. 2011; Yang et al. 2019).

However, the infrageneric classification of *Epimedium* genus remains debatable due to frequent interspecific hybridization and gene introgression. In modern phylogenetic research, chloroplast genomes have been extensively used due to their special advantages such as moderate nucleotide substitution rate, relatively conserved gene sequence and genome structure (Zhang and Li 2011). Therefore, it is still necessary to sequence and assemble chloroplast genomes from more species in order to clarify the intractable phylogenetic relationships within *Epimedium* genus.

In 1884, Franchet (French botanist) published *Epimedium fargesii* Franch. and *Epimedium sutchuenense* Franch. based on the type specimen that Paul Farges (a French missionary) collected in the Chengkou county of Chongqing city (Franchet 1886). *E. sutchuenense* is narrowly distributed in the Daba Mountains (mainly in the Wanyuan County and Qu County of Sichuan province, the Wuxi County, Chengkou County, and Kai County of Chongqing city, and the Shennongjia Forestry District in Hubei province, China) and it is used as “Herba Epimedii” by local people. Specially, *E*. *sutchuenense* is unique among *Epimedium* species for its long-creeping rhizome and the narrowly lanceolate inner sepals which are about as long as the petals (Stearn, 2002). Furthermore, controversies existed all along about whether *E*. *sutchuenense* should be used as ‘Herba Epimedii’ since prenylated flavonoid and its glycosides are nearly absent in *E*. *sutchuenense* (Guo and Xiao 1996; Qin et al. 2020). In this study, we report the first complete chloroplast genome of *E. sutchuenense* and the results will provide useful data for resolving the phylogenetic relationships within *Epimedium* genus.

For this study, the *E. sutchuenense* was sampled from the Shennongjia Forestry District in Hubei province, China (latitude 31.7467 and longitude 110.6709). A specimen and the extracted DNA were deposited at Medicinal Plants Authentication Center, Institute of Medicinal Plant Development, Chinese Academy of Medical Science, Beijing, China (http://www.implad.ac.cn/, collected by Baolin Guo, blguo@implad.ac.cn) under the voucher number B. L. Guo 0437. Genomic DNA was extracted from the fresh leaves of *E. sutchuenense* with the modified CTAB method (Doyle and Doyle 1987). The high-quality DNA was sheared to an average size of 300 bp for library construction using the VAHTSTM Universal DNA Library Pren Kit (ExCell Bio. Biological Technology Co., Ltd, Shanghai, China), and then was sequenced on the Illumina Novaseq 6000 platform (Illumina Inc., San Diego, CA). For assembly, GetOrganelle v1.5 (Jin et al. 2018) was employed to assemble the full length of chloroplast genome sequences with *E. acuminatum* (GenBank accession number: NC_029941) as reference. The annotation of chloroplast genome was conducted through the online program CPGAVAS2 (Shi et al. 2019) and followed by manual correction. The annotated genomic sequence was registered into GenBank with an accession number (MW483087).

The complete chloroplast genome of *E. sutchuenense* was 157,218 bp in length, including two inverted repeat regions (IR_A_ and IR_B_, 25,782 bp) separated by a large single copy region (LSC, 88,575 bp) and a small single copy region (SSC, 17,079 bp). The total GC content was 38.78%, with IR regions having the highest GC content (43.20%), followed by the LSC (37.38%) and SSC region (32.77%). A total of 112 unique genes were identified from the chloroplast genome of *E. sutchuenense*, including 78 protein-coding genes, 30 tRNA genes and 4 rRNA genes. The intron-exon structure analysis showed that a total of 18 genes were found to have introns, among which *pet*B, *pet*D, *rpl*16, *rpl*2, *rpo*C1, *rps*16, *trn*A-UGC, *trn*G-UCC, *trn*I-GAU, *trn*K-UUU, *trn*L-UAA, *trn*V-UAC, *atp*F, *ndh*A and *ndh*B have one intron, while *ycf*3, *rps*12, and *clp*P contain two introns.

To determine the phylogenetic position of *E. sutchuenense*, phylogenetic analysis was performed using the complete chloroplast genome sequences of *E. sutchuenense* and other 10 species downloaded from the NCBI GenBank database. Multiple sequence alignments were generated by using MAFFT v7 (Katoh et al. 2019) and then a Maximum Likelihood (ML) tree was constructed by using IQ-TREE multicore v 2.0.3 (Minh et al. 2020) with *Vancouveria hexandra* (Hook.) C. Morren & Decne as the outgroup (Figure 1). The phylogenetic analysis revealed that *E. sutchuenense* formed a sister relationship with *Epimedium wushanense* T. S. Ying. Our results provided useful information for future research on the evolutionary relationships within the *Epimedium* genus.

**Figure 1.**
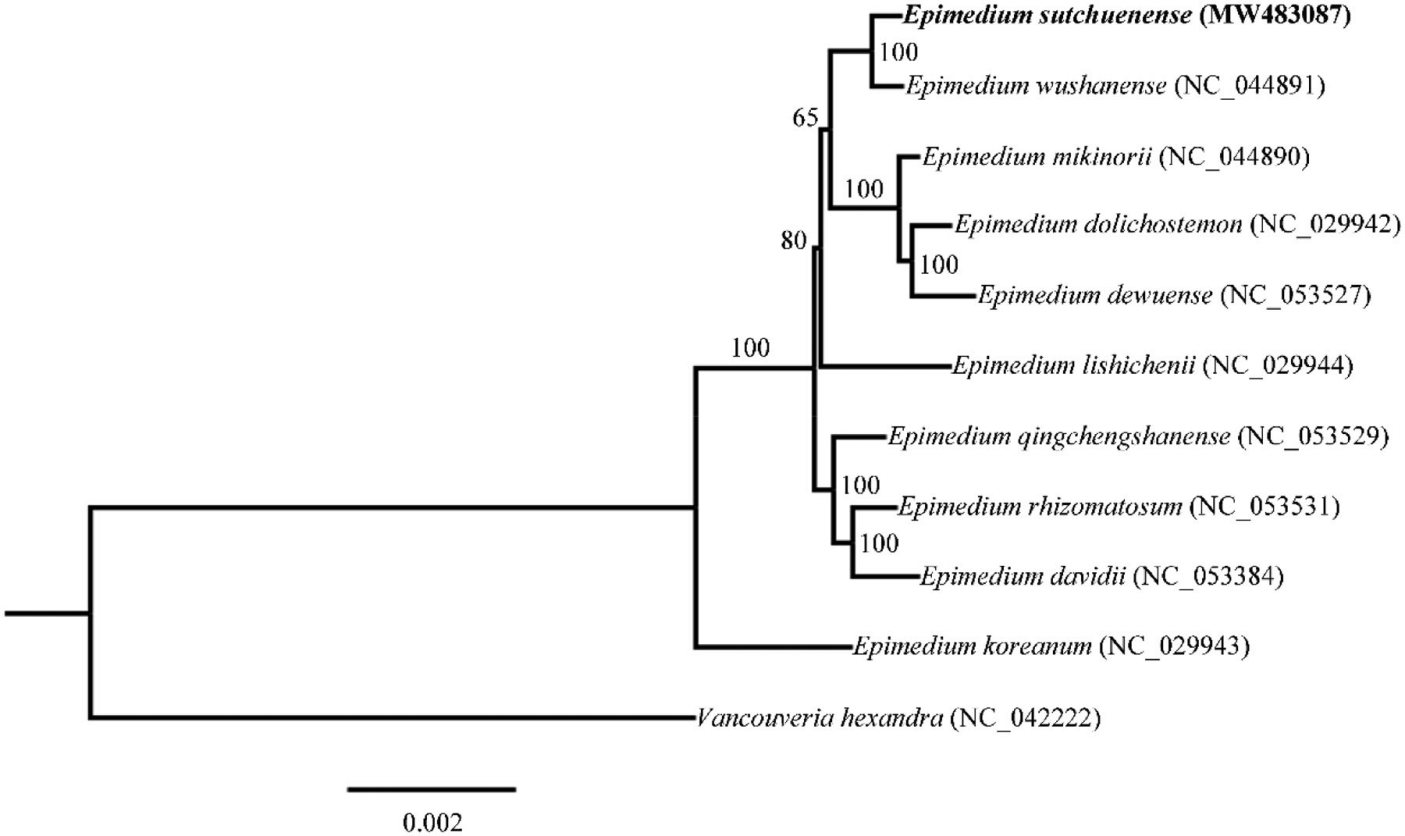
Maximum likelihood (ML) phylogenetic tree based on complete chloroplast genomes of 11 species, with *Vancouveria hexandra* as outgroup. Numbers at nodes represent bootstrap values.

## Data Availability

The genome sequence data that support the findings of this study are openly available in GenBank of NCBI at (https://www.ncbi.nlm.nih.gov/) under the accession no. MW483087. The associated numbers are PRJN751491, SRR15323871, and SAMN20520045, respectively.
